# THE MEDIATION EFFECT OF SOCIAL SUPPORT BETWEEN DEPRESSIVE SYMPTOMS AND LONELINESS IN OLDER ADULTS WITH DIABETES

**DOI:** 10.1093/geroni/igad104.3650

**Published:** 2023-12-21

**Authors:** Emma Cho

**Affiliations:** University of Pennsylvania, Philadelphia, Pennsylvania, United States

## Abstract

The purpose of this study was to examine the relationship between depressive symptoms and loneliness in older adults with diabetes, and to explore the mediating role of social support in this association. This study was developed as a secondary data analysis using data from the National Social Life, Health, and Aging Project in the USA. The sample consisted of 407 older adults aged 50 to 93 years (mean = 64.76, SD=9.03; 50.9% male; 60% married and white). A multi-item survey questionnaire was used to assess loneliness, social support, and depressive symptoms. Spearman correlation analysis was used to examine the relationship between depressive symptoms, social support, and loneliness; the macro PROCESS on SPSS was used to examine the mediating effects of social support on the association between depressive symptoms and loneliness. Despite the significant correlation between depressive symptoms and social support, social support and loneliness, and depressive symptoms and loneliness, social support did not mediate the influence of depressive symptoms on loneliness. The results of this study support the need for increased awareness of depressive symptoms in older adults with diabetes. Depressive symptoms should be treated to alleviate loneliness, and more research is needed to explore how other social factors influence the relationship between depressive symptoms and loneliness, thereby preventing a synergistic effect of depressive symptoms and loneliness.

